# Identification of distinct clinical phenotypes in mechanically ventilated patients with acute brain dysfunction using cluster analysis

**DOI:** 10.1097/MD.0000000000020041

**Published:** 2020-05-01

**Authors:** Vicente Cés Souza-Dantas, Felipe Dal-Pizzol, Cristiane D. Tomasi, Nelson Spector, Márcio Soares, Fernando A. Bozza, Pedro Póvoa, Jorge I. F. Salluh

**Affiliations:** a School of Medicine, Universidade Federal do Rio de Janeiro, Rua Professor Paulo Rocco 255, Cidade universitária, Rio de Janeiro; b Laboratório de Fisiopatologia Experimental, Programa de pós-graduação em ciências da saúde, Universidade do Extremo Sul Catarinense, Avenida Universitária; c Intensive Care Unit, São José Hospital; d São José Hospital Research Center, Rua Coronel Pedro Benedet; e Núcleo de Estudos e Pesquisas em Integralidade e Saúde – NEPIS; f Programa de Pós-Graduação em Saúde Coletiva, Universidade do Extremo Sul Catarinense, Avenida Universitária 1105, Criciúma, SC; g D’or Institute for Research and Education, Rua Diniz Cordeiro 30, Botafogo; h Postgraduation Program, Instituto Nacional de Câncer, Praça Cruz Vermelha 23, Centro; i National Institute of Infectious Disease Evandro Chagas, Oswaldo Cruz Foundation (FIOCRUZ), Rio de Janeiro, Brazil; j Polyvalent Intensive Care Unit, Centro Hospitalar de Lisboa Ocidental, São Francisco Xavier Hospital, Estrada Forte do Alto Duque, Lisbon; k NOVA Medical School, CEDOC, New University of Lisbon, Campo Mártires da Pátria 130, Lisbon, Portugal.

**Keywords:** acute brain dysfunction, cluster analysis, C-reactive protein, critically ill patients

## Abstract

Acute brain dysfunction (ABD) is a frequent and severe syndrome occurring in critically ill patients and early identification of high-risk patients is paramount. In the present analysis, we propose a clinically applicable model for early phenotype identification of ABD at the bedside in mechanically ventilated patients, improving the recognition of patients with prolonged ABD.

Prospective cohort with 629 mechanically ventilated patients in two medical-surgical intensive care units at academic centers. We applied cluster analysis to identify phenotypes using clinical and biological data. We then tested the association of phenotypes and its respective clinical outcomes. We performed a validation on a new cohort of patients select on subsequent patients admitted to the participants intensive care units.

A model with 3 phenotypes best described the study population. A 4-variable model including medical admission, sepsis diagnosis, simplified acute physiologic score II and basal serum C-reactive protein (CRP) accurately classified each phenotype (area under curve 0.82; 95% CI, 0.79–0.86). Phenotype A had the shorter duration of ABD (median, 1 day), while phenotypes B and C had progressively longer duration of ABD (median, 3 and 6 days, respectively; *P* < .0001). There was an association between the duration of ABD and the baseline CRP levels and simplified acute physiology score II score (sensitivity and specificity of 80%). To increase the sensitivity of the model, we added CRP kinetics. By day 1, a CRP < 1.0 times the initial level was associated with a shorter duration of ABD (specificity 0.98).

A model based on widely available clinical variables could provide phenotypes associated with the duration of ABD. Phenotypes with longer duration of ABD (phenotypes B and C) are characterized by more severe inflammation and by significantly worse clinical outcomes.

## Abstract 2

Supplemental Digital Content is available in the text

## 1 Introduction

Acute brain dysfunction (ABD), defined as the presence of coma or delirium, is a severe and frequent syndrome in patients admitted to the intensive care unit (ICU).^[1–3]^ Numerous studies demonstrate that ABD is associated with increased mortality, hospital length of stay and costs, as well as, long-term cognitive impairment.^[2–7]^

Accurate and early identification of high-risk patients may provide relevant information to guide clinical interventions, especially those aiming at a preemptive or preventive approach of delirium. Currently available ICU delirium prediction models use variables at ICU admission and at 24 hours to stratify ICU patients for the risk of delirium occurrence with a high discriminative power.^[8,9]^ However, although they can predict delirium during ICU stay, they are not able to predict the duration of delirium or coma. Recent data confirmed that more than the occurrence of delirium, the duration and severity of coma and delirium duration are the major predictors of poor outcome.^[10–13]^

Clinical management could potentially be improved by using reliable phenotypes of ABD based on clinical risk factors and biomarkers. However, there are few studies using this approach for ICU patients.

Cluster analysis is a multivariate statistical method that identifies groups of cases according to similarity on certain well-accepted characteristics (phenotype) of a specific disorder without the constraint of an a priori diagnostic system.^[14]^ It has previously been used to identify profiles of non-critically ill individuals with delirium.^[15,16]^ However, to the best of our knowledge, cluster analysis has never been used to find groupings of factors (clinical data) that fall into distinct phenotypes that are associated with the duration of ABD, trying to predict and classify its duration.

In the present analysis, we proposed a clinically applicable model which uses easily available clinical and biological (biomarkers) information for early phenotype identification of ABD at the bedside in mechanically ventilated patients that may have impact for identifying patients with longer duration of ABD.

## 2 Methods

### 2.1 Design and setting

**Figure 1:**
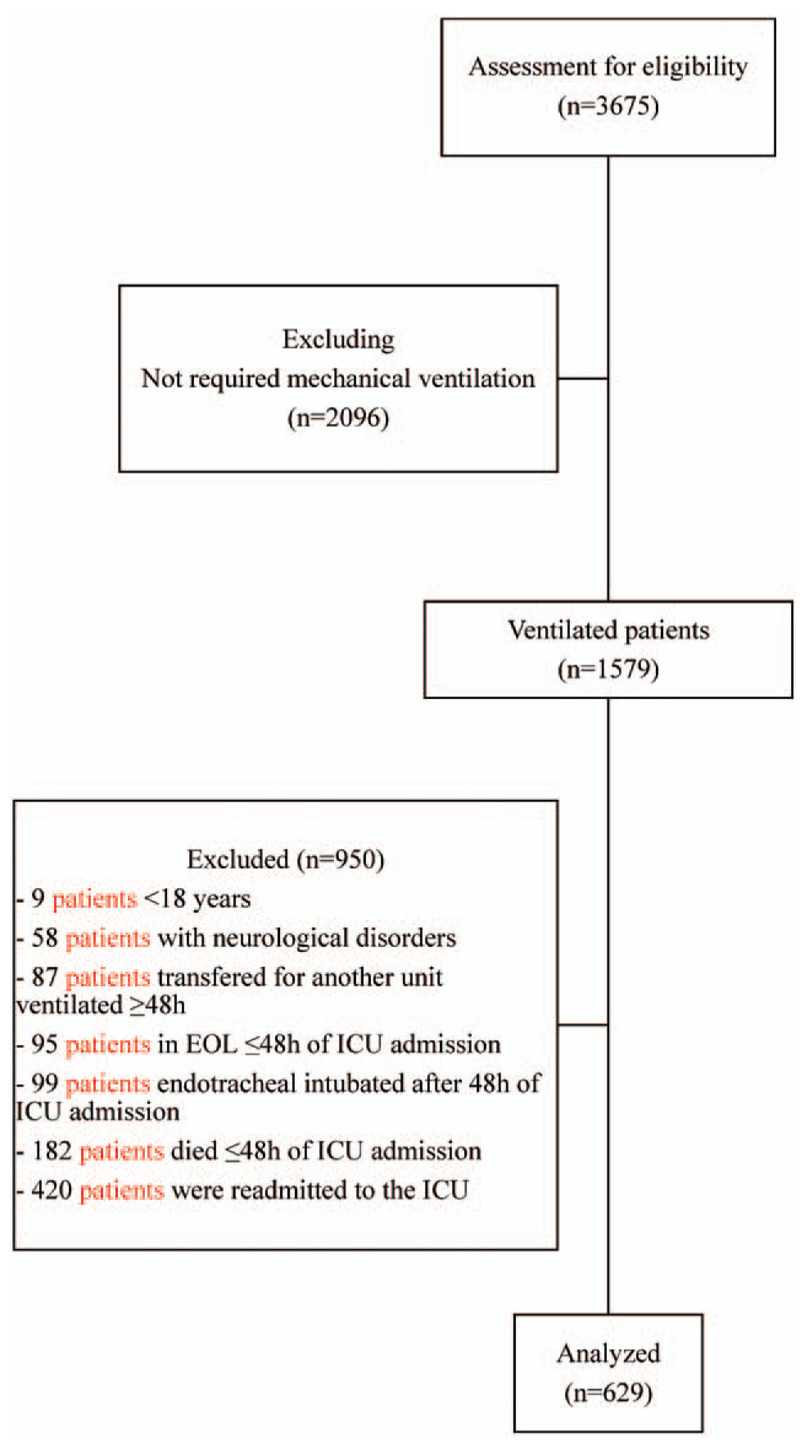
Inclusion flowchart. EOL = end of life care, ICU = intensive care unit.

This was a prospective cohort study performed in the ICUs of Instituto Nacional de Câncer (INCA), Rio de Janeiro and the São José Hospital, Criciúma, Brazil. INCA's ICU is a twenty-bed medical-surgical unit specialized in the care of patients with cancer,^[17]^ with the exception of bone marrow transplant patients. São José Hospital is a university hospital with a twenty-bed general medical-surgical ICU. Briefly, during the study period (November 2009 to September 2013), we evaluated every adult patient (≥ 18 years) who required ICU admission and mechanical ventilation (MV) >48 hours. Patients ventilated > 24 hours prior to ICU admission, patients ventilated after 48 hours of ICU admission and readmissions were excluded. Patients with blindness, deafness or incapable of speaking Portuguese, moribund patients (expected to die in < 48 hours), as well as patients with previous neurologic disorders were also excluded (Fig. 1).

### 2.2 Definitions, selection of participants and data collection

The Ethics Committees of the Instituto Nacional de Câncer in Rio de Janeiro and of São José Hospital in Criciúma approved the study (Number: 144/2009; and Number: 25915513.3.0000.0119, respectively) and all patients or proxies gave written informed consent, without any refusal.

We collected demographic, clinical and laboratory data using standardized case report forms that included comorbidities, the Simplified Acute Physiology Score (SAPS) II^[18]^ and the Sequential Organ Failure Assessment (SOFA)^[19]^ without Glasgow Coma Scale component. Duration of the MV, ICU, hospital and 90-day mortality rates from any cause were also assessed. All patients were followed up until death or hospital discharge.

The use of sedatives was registered. The level of arousal was measured using the Portuguese version of Richmond Agitation and Sedation Scale (RASS).^[20]^ Coma was defined as a RASS of minus 4 (responsive only to physical stimulus) or minus 5 (unresponsive to physical stimulus).^[21]^ Delirium was diagnosed with the Brazilian-Portuguese version of confusion assessment method (CAM)-ICU.^[22]^ The participating ICUs had sedation protocols according to the best practices at the time of the study,^[23]^ which included the daily suspension of sedatives in all patients. Patients were sedated titrated to a RASS^[21]^ ideal target between 0 and −1, unless the consultant intensivist responsible for clinical management decided a deeper level of sedation on a given day. RASS was assessed every 4 hour. We did not use a formal pain score and analgesics were titrated according to the bedside nurse's judgment of the patient's level of comfort and pain as assessed by local protocols. Patients were assessed for delirium every morning 3 hours after daily sedation/analgesic holds, by a trained investigator during the first 8 days of ICU stay.

C-reactive protein (CRP) levels were measured at ICU admission and during 7 consecutive days. For purposes of the analysis, Day 0 (D0) was defined as the day of ICU admission. We assessed a new variable, CRP-ratio, which was calculated as the day's CRP concentration divided by the D0 CRP concentration. Serum CRP level was determined with the Roche Cobas Integra 800 analyzer (Roche Diagnostic, Indianapolis, IN).

The main outcome of interest was the duration of ABD. Other measured outcomes were CRP, organ dysfunctions assessed by SOFA, MV duration and also ICU, hospital and 90-day mortality.

### 2.3 Data processing and statistical analysis

Baseline clinical data were considered as class-defining variables in a two-steps hierarchical cluster analysis that was carried out using Ward's method, applying squared Euclidean Distance as the similarity measure.^[24]^ The classification was conducted without consideration of clinical outcomes. Clinical variables included in the model were age, gender, Charlson comorbidity index, SAPS II score, SOFA score, medical admission, diagnosis of sepsis at admission, CRP at day 0 and the use of sedatives. They are presented in Table 1.

**Table 1:**
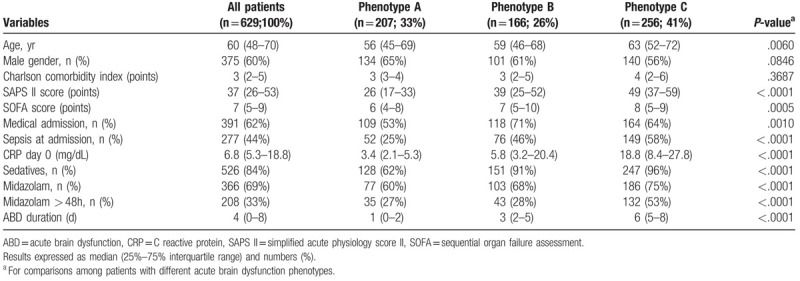
Demographic and clinical variables of patients according to the acute brain dysfunction phenotype.

The aim of this statistical method was to find relatively homogeneous clusters of cases based on measured characteristics. The log-likelihood method was used to determine inter-subject distance and specific classification of participants. The model was produced using the Schwarz Bayesian criterion. Many different models were subsequently produced using a different number of predetermined classes.

Once the number of classes was determined, the association between classes and clinical outcomes (ICU, hospital and 90-day mortality; duration of MV and MV free-days) was tested. We used standard descriptive statistics and reported continuous variables as median [25%–75% interquartile range]. Comparisons between groups were performed with two-tailed unpaired Student *t*-test, one-way ANOVA, Mann-Whitney *U* or Kruskal-Wallis *H* tests for continuous variables according to data distribution. Fisher exact test and Chi-square test were used to carry out comparisons between categorical variables as appropriate.

We subsequently tested how well a smaller number of variables could identify the predetermined classes. We used a forward stepwise modeling and we identified 4 variables that contributed independently to class assignment. We also tested the performance characteristics of these models. The model was validated on a new cohort of ICU patients.

In an attempt to improve the model's ability to sort the classes, we used CRP kinetics as a second classification step to correctly define the classes. The CRP kinetics was assessed for its association with the duration of ABD by conventional bivariate correlation and a linear regression model.

We performed time-dependent analysis of different variables with general linear model, univariate, repeated-measures analysis using a split-plot design approach.^[25]^

One investigator (V.C.S.D) performed data entry and assessed data consistency by a rechecking procedure in a random sample of patients. We carried out all statistical analyses using the SPSS 23.0 software package (Chicago, Illinois) and Prism 6.0 (Graphpad).

## 3 Results

### 3.1 Cluster analysis: identification of number of phenotypes

We began by fitting cluster models ranging from one to five classes. The Bayesian information criterion (BIC) decreased as the number of classes increased, suggesting that the addition of subsequent classes could be adding additional information to the model (Table 2). Entropy in models with three and four classes was > 0.80, indicating strong separation between the classes. Using the likelihood ratio test, a 3-class model was a significant improvement over a 2-class model (*P*=.04). In our patient population additional classes did not provide a statistically significant improvement. We retained a final three-class model based on these results.

**Table 2:**
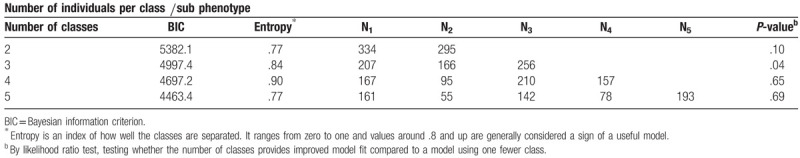
Fit class cluster.

The three-class model estimated 207 subjects in Class 1 (33%), 166 subjects in Class 2 (26%) and 256 subjects in Class 3 (41%). Because of the high average cluster class probabilities of class assignment, there is minimal loss of information. We will subsequently refer to class 1, 2, and 3 as phenotypes A, B and C.

### 3.2 Phenotypes have distinct characteristics and clinical outcomes

Only 2 (0.0003%) of the patients did not present any sort of ABD (coma or delirium during the study period). Sixty-nine (11%) patients had only coma and 125 (20%) had only delirium, being that 48 (38%) of these patients presented only short-term delirium, throughout the study period. The rest of the patients fluctuated between coma, delirium and periods of normal cognition.

The baseline clinical and biological characteristics among the 3 phenotypes were different on demographics, clinical characteristics and severity of illness (Table 1). A significantly longer duration of ABD was observed in phenotype B and C [median, 3 and 6 days, respectively] as compared to A [median, 1 day; *P* < .0001]. Patients with phenotypes B and C were older, sicker, as expressed by higher baseline SAPS II, and sepsis at ICU admission. They also showed significantly longer ICU and hospital length of stay, had higher ICU, hospital and 90-day mortality and exhibited longer duration of MV (Supplemental Digital Content 1).

### 3.3 A four-variable model can accurately identify distinct ABD phenotypes

The cohort of the study consisted of 629 patients that were included in the development dataset. Another 200 patients subsequent patients admitted to the participants ICUs (January 2016 to March 2018) with the same inclusion criteria of the patients in the development dataset were included in the validation dataset. The demographic characteristics of both groups were comparable (see Supplemental Digital Content 2).

Using forward stepwise modeling, the four variables that contributed most to phenotype assignment (in order of contribution) were SAPS II, medical admission, diagnosis of sepsis and basal serum CRP. We then measured the accuracy of models using the top four variables for phenotype identification in the development and in the validation dataset [see Supplemental Digital Content 3 andd 4]. The “top four” variable model had an area under the curve (AUC) of 0.82 (95% CI, 0.79–0.86) in the development dataset and 0.81 (95% CI, 0.77–0.87) in the validation dataset. Using Youden index, the optimal sensitivity and specificity of the four-variable model was 75% and 73%, respectively. The parameter estimates for the four-variable model are listed in Table 3.

**Table 3:**
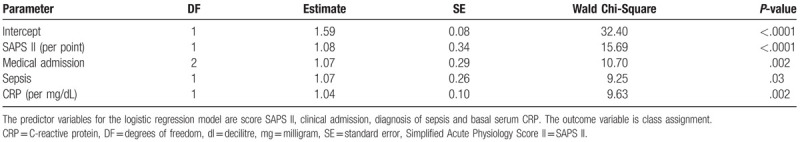
Logistic regression parameter estimates for the 4-variable model.

In order to improve the accuracy of the model, we performed a time-dependent analysis of the CRP from day 0 to day 7 comparing the distinct clusters. The analysis showed that increases in CRP levels on day 1 were only present in patients with longer ABD duration (clusters B and C), thereafter, there was a progressive decrease in CRP levels (*P* < .0001; see Supplemental Digital Content 5).

**Figure 2:**
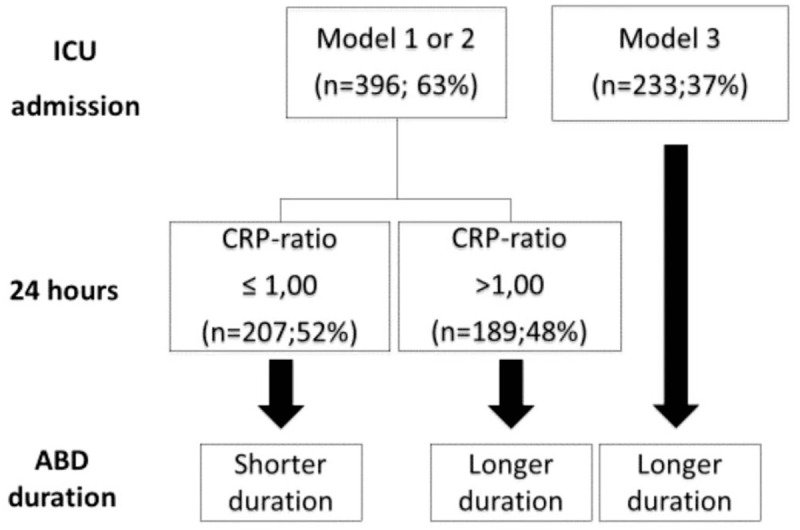
Clinical algorithm of acute brain dysfunction duration. ABD = acute brain dysfunction, CRP = C-reactive protein, ICU = intensive care unit.

Then, we proposed a clinical algorithm containing the 4-variable model at ICU admission, associated with the variation in the D1 CRP-ratio, allowing us to help in the early identification of patients with longer duration of ABD (Fig. 2). Patients with medical admission, sepsis diagnosis at ICU admission, basal CRP > 18.8mg/dL, and score SAPS II > 53 points (Model 3), always (n = 233; 100%) had longer duration of ABD, compatible with the duration of ABD seen in patients with Phenotype C.

However, patients with clinical characteristics compatible with Models 1 and 2 had variable duration of ABD. Using the D1 CRP-ratio, patients with the ratio < 1.0 (n = 207; 52%) had shorter duration of ABD (compatible with the duration of ABD seen in patients with Phenotype A), while the patients with the ratio > 1.0 (n = 189;49%) had longer duration of ABD (compatible with the duration of ABD seen in patients with Phenotype B).

### 3.4 SAPS II and the duration of ABD

The higher the SAPS II, the longer the duration of ABD (*P* < .0001). Phenotype A patients had the lowest SAPS II (median, 26 points), while patients with phenotypes B and C had the higher SAPS II (median, 39 and 49 points, respectively; *P* < .0001). On admission, SAPS II greater than 34 points were associated with longer duration of ABD, with sensitivity and specificity of 80% (positive likelihood ratio 3.98; negative likelihood ratio 0.25). With each 8-point increase in the SAPS II, the duration of ABD increased by 0.5 days (*P* < .0001) and the risk of longer ABD duration increased by 8% (odds ratio 1.08; 95% CI, 1.01–1.16; *P* < .0001).

### 3.5 CRP and the duration of ABD

A higher baseline CRP level, was a associated with longer the duration of ABD (*P* < 0.0001). As expected, phenotype A patients had the lowest baseline CRP levels (median, 3.4mg/dL), while patients with phenotypes B and C, had the higher CRP levels (median, 5.8mg/dL and 18.8mg/dL, respectively; *P* < .0001). At baseline, CRP values greater than 5.3 mg/dL were associated with longer ABD duration with both sensitivity and specificity of 75% (positive likelihood ratio 3.25; negative likelihood ratio 0.36). With each 10.5 mg/dL increase in baseline CRP, the duration of ABD was increased by 1.7 days (*P* < .0001) and the risk of longer ABD duration was increased by 7% (odds ratio, 1.07; 95% CI 1.04–1.10). The longer duration of ABD related to baseline CRP levels and the CRP kinetics was clear regardless of the use of sedatives.

## 4 Discussion

This study provides evidence that there are at least 3 phenotypes of ABD, with different clinical characteristics, biomarker profiles, and clinical outcomes when critically ill mechanically ventilated patients are evaluated. This 4-variable model that incorporates clinical admission, diagnosis of sepsis, SAPS II and basal serum CRP easily identifies the 3 ABD phenotypes. We also found a qualitative relation between ABD phenotype, as defined by cluster analysis and early CRP kinetics, in which persistent systemic inflammation seems to be associated with longer duration of brain dysfunction. To the best of our knowledge this is the first study evaluating the early assessment of CRP kinetics as a predictor of the duration of ABD in critically ill patients.

Currently specific intervention that could effectively prevent or treat ABD in unselected patients are scarce.^[26]^ However, the early identification of patients with high risk of longer duration of ABD, those with phenotypes B and C, could lead the attending physician to a change in the prescription of sedatives away from drugs with a well-known risk of inducing or aggravating ABD, like benzodiazepines. And in the future, we could speculate that these phenotypes could be useful in the selection of more personalized approach of ABD in the clinical setting or in trials.

The brain is frequently involved by the systemic inflammation response in critical illness, and overexpression of proinflammatory cytokines is directly associated with brain dysfunction.^[27]^ Few studies have evaluated the usefulness of CRP^[28–32]^ both in the diagnosis and in the prediction of patients with high risk of developing ABD. Most published studies^[28–31]^ evaluated the discriminative power for brain dysfunction diagnosis of a single determination of CRP.

In previous studies of critically ill patients with severe infections^[33,34]^ there was a clear association of patterns of CRP-ratio response with outcomes, such as mortality, organ failures and response to antibiotic therapy. In the present study, we used the CRP-ratio to evaluate its role as an early marker of the duration of ABD.

Our study has several strengths. First, since clinical outcomes were not considered as class-defining variables, the strengths and consistency of the associations between phenotypes and clinical outcomes are striking. Second, we evaluated a large cohort of MV patients evaluating the course of CRP. Therefore, all eligible patients were included, independently of the presence of comorbidities or underlying diagnoses, which makes our study population more representative of ICU patients. In addition, we used a biomarker that is fast, cheap, validated in clinical practice, and routinely available. Finally, the sequential use of clinical clustering and evaluation of CRP kinetics provided a two-step approach that further refined the early assessment of persistent ABD.

This study also has several limitations. Although the sample size provided an adequate statistical power to identify 3 subgroups of patients, a larger sample could be more representative of MV critically ill patients. Eventually, larger number and more homogeneous clusters can emerge with a higher number of participants. Moreover, the study was implemented in only two hospitals, and although they are likely to be representative of modern in-patient medical centres, further work replicating these findings elsewhere are warranted. Additionally, we provide no data on the different interventions to prevent and treat delirium. The addition of this information in future studies could be valuable to identify phenotypes that are more responsive to specific interventions. The incidence and severity of ABD is markedly influenced by the use of sedative agents. Owing to the observational nature of our study changes in the sedative agents’ prescription was not controlled, and we acknowledge that it could constitute a confounding factor.

Also, as brain dysfunction was evaluated only once daily and, as ABD is a fluctuating syndrome, some diagnoses may have been missed. Also, the majority of the participants had ABD, important clinical features, such as motor subtypes, and persistence of subsyndromal symptoms were not assessed. Finally, long-term follow-up was not performed and therefore, we cannot draw conclusions on the impact of these 3 phenotypes on long-term cognitive function of these patients.

## 5 Conclusions

In summary, the use of simple and widely available clinical variables supports the presence of 3 distinct ABD phenotypes. The application of the 4-variable model at ICU admission, associated with the variation in the D1 CRP-ratio, allowed us to propose an accurate model that helps in the early identification of patients with longer duration of ABD. We believe that current results may have an impact on the effectiveness of identification of patients with prolonged ABD, assisting in clinical practice and selection criteria for pharmacological intervention trials.

## Author contributions

VCSD, FDP, CDT, NS, MS, FAB, PP and JIFS contributed substantially to the study design, data analysis and interpretation, and the writing of the manuscript. All authors read and approved the final manuscript.

**Conceptualization:** Vicente SOUZA-DANTAS, Felipe Dal-Pizzol, Cristiane D. Tomasi, Nelson Spector, Márcio Soares, Fernando A. Bozza, Pedro Póvoa, Jorge I. F. Salluh.

**Data curation:** Vicente SOUZA-DANTAS, Felipe Dal-Pizzol, Cristiane D. Tomasi, Márcio Soares, Pedro Póvoa, Jorge I. F. Salluh.

**Formal analysis:** Vicente SOUZA-DANTAS, Felipe Dal-Pizzol, Cristiane D. Tomasi, Márcio Soares, Fernando A. Bozza, Pedro Póvoa, Jorge I. F. Salluh.

**Funding acquisition:** Vicente SOUZA-DANTAS, Felipe Dal-Pizzol, Cristiane D. Tomasi, Jorge I. F. Salluh.

**Investigation:** Vicente SOUZA-DANTAS, Felipe Dal-Pizzol, Cristiane D. Tomasi, Márcio Soares, Pedro Póvoa, Jorge I. F. Salluh.

**Methodology:** Vicente SOUZA-DANTAS, Felipe Dal-Pizzol, Cristiane D. Tomasi, Nelson Spector, Márcio Soares, Fernando A. Bozza, Pedro Póvoa, Jorge I. F. Salluh.

**Project administration:** Vicente SOUZA-DANTAS, Felipe Dal-Pizzol, Cristiane D. Tomasi, Márcio Soares, Fernando A. Bozza, Jorge I. F. Salluh.

**Resources:** Vicente SOUZA-DANTAS, Felipe Dal-Pizzol, Cristiane D. Tomasi, Márcio Soares, Jorge I. F. Salluh.

**Software:** Vicente SOUZA-DANTAS, Felipe Dal-Pizzol, Cristiane D. Tomasi, Márcio Soares, Jorge I. F. Salluh.

**Supervision:** Vicente SOUZA-DANTAS, Felipe Dal-Pizzol, Cristiane D. Tomasi, Nelson Spector, Márcio Soares, Pedro Póvoa, Jorge I. F. Salluh.

**Validation:** Vicente SOUZA-DANTAS, Felipe Dal-Pizzol, Cristiane D. Tomasi, Márcio Soares, Pedro Póvoa, Jorge I. F. Salluh.

**Visualization:** Vicente SOUZA-DANTAS, Felipe Dal-Pizzol, Cristiane D. Tomasi, Márcio Soares, Jorge I. F. Salluh.

**Writing – original draft:** Vicente SOUZA-DANTAS, Felipe Dal-Pizzol, Cristiane D. Tomasi, Nelson Spector, Márcio Soares, Fernando A. Bozza, Pedro Póvoa, Jorge I. F. Salluh.

**Writing – review and editing:** Vicente SOUZA-DANTAS, Felipe Dal-Pizzol, Cristiane D. Tomasi, Nelson Spector, Márcio Soares, Fernando A. Bozza, Pedro Póvoa, Jorge I. F. Salluh.

## Supplementary Material

Supplemental Digital Content

## Supplementary Material

Supplemental Digital Content

## Supplementary Material

Supplemental Digital Content

## Supplementary Material

Supplemental Digital Content

## Supplementary Material

Supplemental Digital Content

## References

[1] Gofton TE Young GB Sepsis-associated encephalopathy. Nat Rev Neurol 2012;8:557–66. 22986430 10.1038/nrneurol.2012.183

[2] Ely EW Gautam S Margolin R The impact of delirium in the intensive care unit on hospital length of stay. Intensive Care Med 2001;27:1892–900. 11797025 10.1007/s00134-001-1132-2 PMC7095464

[3] Ely EW Shintani A Truman B Delirium as a predictor of mortality in mechanically ventilated patients in the intensive care unit. JAMA 2004;291:1753–62. 15082703 10.1001/jama.291.14.1753

[4] Milbrandt EB Deppen S Harrison PL Costs associated with delirium in mechanically ventilated patients. Crit Care Med 2004;32:955–62. 15071384 10.1097/01.ccm.0000119429.16055.92

[5] Pandharipande PP Girard TD Jackson JC Long-term cognitive impairment after critical illness. N Engl J Med 2013;369:1306–16. 24088092 10.1056/NEJMoa1301372 PMC3922401

[6] Jackson JC Pandharipande PP Girard TD Depression, post-traumatic stress disorder, and functional disability in survivors of critical illness in the BRAIN-ICU study: a longitudinal cohort study. Lancet Respir Med 2014;2:369–79. 24815803 10.1016/S2213-2600(14)70051-7 PMC4107313

[7] Hopkins RO The brain after critical illness: effect of illness and aging on cognitive function. Crit Care 2013;17:116. 23384320 10.1186/cc11913 PMC4057412

[8] van den Boogaard M Pickkers P Slooter AJ Development and validation of PRE-DELIRIC (PREdiction of DELIRium in ICu patients) delirium prediction model for intensive care patients: observational multicentre study. BMJ 2012;344:e420. 22323509 10.1136/bmj.e420 PMC3276486

[9] Wassenaar A van den Boogaard M van Achterberg T Multinational development and validation of an early prediction model for delirium in ICU patients. Intensive Care Med 2015;41:1048–56. 25894620 10.1007/s00134-015-3777-2 PMC4477716

[10] Almeida ICT Soares M Bozza Fa The impact of acute brain dysfunction in the outcomes of mechanically ventilated cancer patients. Plos One 2014;9:e85332. 24465538 10.1371/journal.pone.0085332 PMC3899009

[11] Klouwenberg PMCK Zaal IJ Spitoni C The attributable mortality of delirium in critically ill patients: prospective cohort study. BMJ 2014;349:g6652. 25422275 10.1136/bmj.g6652 PMC4243039

[12] Shehabi Y Bellomo R Reade MC Early intensive care sedation predicts long-term mortality in ventilated critically ill patients. Am J Respir Crit Care Med 2012;186:724–31. 22859526 10.1164/rccm.201203-0522OC

[13] Gunther ML Morandi A Krauskopf E The association between brain volumes, delirium duration, and cognitive outcomes in intensive care unit survivors: the VISIONS cohort magnetic resonance imaging study. Crit Care Med 2012;40:2022–32. 22710202 10.1097/CCM.0b013e318250acc0 PMC3697780

[14] Everitt BS Landau S Leese M Cluster Analysis. Wiley, 5th ed. London: 2011.

[15] Lagarto L Cerejeira J Identification of sub-groups in acutely ill elderly patients with delirium: a cluster analysis. Int Psychogeriatr 2016;28:1283–92. 26972383 10.1017/S1041610216000302

[16] Sepulveda E Farnco JG Trzepacz PT Delirium diagnosis defined by cluster analysis of symptoms versus diagnosis by DSM and ICD criteria: diagnostic accuracy study. BMC Psychiatry 2016;16:167. 27229307 10.1186/s12888-016-0878-6 PMC4882791

[17] Soares M Salluh JIF Spector N Characteristics and outcomes of cancer patients requiring mechanical ventilatory support for 24 hrs. Crit Care Med 2005;33:520–6. 15753742 10.1097/01.ccm.0000155783.46747.04

[18] Le Gall JR Lemeshow S Saulnier F A new Simplified Acute Physiology Score (SAPS II) based on a European/North American multicenter study. JAMA 1993;270:2957–63. 8254858 10.1001/jama.270.24.2957

[19] Vincent JL Moreno R Takala J The SOFA (Sepsis-related Organ Failure Assessment) score to describe organ dysfunction/failure. On behalf of the working group on Sepsis-Related Problems of the European Society of Intensive Care Medicine. Intensive Care Med 1996;22:707–10. 8844239 10.1007/BF01709751

[20] Nassar Junior AP Pires Neto RC de Figueiredo WB Validity, reliability and applicability of Portuguese versions of sedation-agitation scales among critically ill patients. Sao Paulo Med J 2008;126:215–9. 18853029 10.1590/S1516-31802008000400003 PMC11025981

[21] Ely EW Truman B Shintani A Monitoring sedation status over time in ICU patients: reliability and validity of the Richmond Agitation-Sedation Scale (RASS). JAMA 2003;289:2983–91. 12799407 10.1001/jama.289.22.2983

[22] Gusmao-Flores D Salluh JI Dal-Pizzol F The validity and reliability of the Portuguese versions of three tools used to diagnose delirium in critically ill patients. Clinics (Sao Paulo) 2011;66:1917–22. 22086522 10.1590/S1807-59322011001100011 PMC3203964

[23] Girard TD Kress JP Fuchs BD Efficacy and safety of a paired sedation and ventilator weaning protocol for mechanically ventilated patients in intensive care (Awakening and Breathing Controlled trial): a randomized controlled trial. Lancet 2008;371:126–34. 18191684 10.1016/S0140-6736(08)60105-1

[24] Hagen DR Tidor B Efficient Bayesian estimates for discrimination among topologically different systems biology models. Mol Biosyst 2015;11:574–84. 25460000 10.1039/c4mb00276h

[25] Monti MM Statistical analysis of fMRI time-series: a critical review of the GLM approach. Front Hum Neurosci 2011;5:28. 21442013 10.3389/fnhum.2011.00028 PMC3062970

[26] Serafim RB Bozza FA Soares M Pharmacologic prevention and treatment of delirium in intensive care patients: a systematic review. J Crit Care 2015;30:199–807. 10.1016/j.jcrc.2015.04.005 25957498

[27] Cerejeira J Firmino H Vaz-Serra A The neuroinflammatory hypothesis of delirium. Acta Neuropathol 2010;119:737–54. 20309566 10.1007/s00401-010-0674-1

[28] Macdonald A Adamis D Treloar A C-reactive protein levels predict the incidence of delirium and recovery from it. Age Ageing 2007;36:222–5. 17114198 10.1093/ageing/afl121

[29] Lemstra AW Kalisvaart KJ Vreeswijk R Pre-operative inflammatory markers and the risk of postoperative delirium in the elderly patients. Int J Geriatr Psychiatry 2008;23:943–8. 18481319 10.1002/gps.2015

[30] Pol RA van Leeuwen BL Izaks GJ C-reactive protein predicts postoperative delirium following vascular surgery. Ann Vasc Surg 2014;28:1923–30. 25017770 10.1016/j.avsg.2014.07.004

[31] McGrane S Girard TD Thompson JL Procalcitonin and C-reactive protein level at admission as predictors of duration of acute brain dysfunction in critically ill patients. Crit Care 2011;15:R78. 21366899 10.1186/cc10070 PMC3219330

[32] Zhang Z Pan L Deng L Prediction of delirium in critically ill patients with with elevated C-reactive protein. J Crit Care 2014;29:88–92. 24120090 10.1016/j.jcrc.2013.09.002

[33] Povoa P Coelho L Almeida E C-reactive protein as a marker of ventilator-associated pneumonia resolution: a pilot study. Eur Respir J 2005;25:804–12.15863636

[34] Povoa P Coelho L Almeida E Pilot study evaluating C-reactive protein levels in the assessment of response to treatment of severe bloodstream infection. Clin Infect Dis 2005;40:1855–7. 15909277 10.1086/430382

