# Supplementary material for: Identification of distinct clinical phenotypes in mechanically ventilated patients with acute brain dysfunction using cluster analysis
Source: Medicine (Baltimore). 2020 May 1;99(18):e20041. doi: 10.1097/MD.0000000000020041 (PMC7440320; doi:10.1097/MD.0000000000020041)
Supplement: Supplemental Digital Content [file medi-99-e20041-s001.docx]

| Variables | All patients (n=629;100%) | Phenotype A  (n=207; 33%) | Phenotype B (n=166; 26%) | Phenotype C (n=256; 41%) | *P*-value^a^ |
| --- | --- | --- | --- | --- | --- |
| MV duration (days) | 8 (4-15) | 3 (2-6) | 6 (4-9) | 15 (10-21) | <0.0001 |
| MV free- days | 2 (0-7) | 4 (1-8) | 2 (0-6) | 1 (0-5) | 0.0236 |
| LOS before ICU (days) | 3 (1-9) | 2 (0-9) | 3 (1-7) | 3 (1-10) | 0.5177 |
| ICU LOS (days) | 12 (7-21) | 8 (4-14) | 9 (5-15) | 19 (13-27) | <0.0001 |
| Hospital LOS (days) | 21 (11-34) | 14 (7-24) | 17 (9-27) | 21 (11-34) | <0.0001 |
| ICU mortality,  n (%) | 295 (47%) | 76 (37%) | 84 (51%) | 135 (53%) | 0.0015 |
| Hospital mortality,  n (%) | 357 (57%) | 84 (41%) | 101 (61%) | 172 (67%) | <0.0001 |
| 90-day mortality,  n (%) | 381 (61%) | 90 (43%) | 104 (63%) | 187 (73%) | <0.0001 |

**Supplemental Digital Content 1. Outcomes of patients according to the acute brain dysfunction phenotype**

^a^For comparisons among patients with different acute brain dysfunction phenotypes.

MV – mechanical ventilation; LOS – length of stay; ICU - intensive care unit.

Results expressed as median (25%–75% interquartile range) and numbers (%).
