# Supplementary material for: Identification of distinct clinical phenotypes in mechanically ventilated patients with acute brain dysfunction using cluster analysis
Source: Medicine (Baltimore). 2020 May 1;99(18):e20041. doi: 10.1097/MD.0000000000020041 (PMC7440320; doi:10.1097/MD.0000000000020041)
Supplement: Supplemental Digital Content [file medi-99-e20041-s002.docx]

| Variables | Development dataset  (n=629;100%) | Validation dataset  (n=200; 100%) |
| --- | --- | --- |
| Age (years) | 60 (48-70) | 55 (44-76) |
| Male gender,  n (%) | 375 (60%) | 132 (66%) |
| Charlson comorbidity index (points) | 3 (2-5) | 4 (3-4) |
| SAPS II score (points) | 37 (26-53) | 36 (27-53) |
| SOFA score (points) | 7 (5-9) | 7 (5-9) |
| Medical admission,  n (%) | 391 (62%) | 124 (62%) |
| Sepsis at admission,  n (%) | 277 (44%) | 98 (49%) |
| ABD duration (days) | 4 (0-8) | 3 (0-4) |
| CRP day 0 (mg/dL) | 6.8 (5.3-18.8) | 6.2 (4.9-24.1) |
| Sedatives,  n (%) | 526 (84%) | 140 (70%) |
| Midazolam,  n (%) | 366 (69%) | 100 (71%) |
| Midazolam >48h, n (%) | 208 (33%) | 27 (27%) |
| MV duration (days) | 8 (4-15) | 8 (6-11) |
| MV free- days | 2 (0-7) | 4 (1-8) |
| LOS before ICU (days) | 3 (1-9) | 3 (1-7) |
| ICU LOS (days) | 12 (7-21) | 16 (12-22) |
| Hospital LOS (days) | 21 (11-34) | 23 (16-33) |
| ICU mortality,  n (%) | 295 (47%) | 84 (42%) |
| Hospital mortality,  n (%) | 357 (57%) | 96 (48%) |
| 90-day mortality,  n (%) | 381 (61%) | 98 (49%) |

ABD – acute brain dysfunction; SAPS II - Simplified Acute Physiology

Score II; SOFA - Sequential Organ Failure Assessment;

CRP – C reactive protein; MV – mechanical ventilation; LOS – length of

stay; ICU - intensive care unit.

Results expressed as median (25%–75% interquartile range) and numbers

(%)
