# Supplementary material for: Identification of distinct clinical phenotypes in mechanically ventilated patients with acute brain dysfunction using cluster analysis
Source: Medicine (Baltimore). 2020 May 1;99(18):e20041. doi: 10.1097/MD.0000000000020041 (PMC7440320; doi:10.1097/MD.0000000000020041)
Supplement: Supplemental Digital Content [file medi-99-e20041-s003.docx]

|  | **Model 1**  (no medical admission;  no sepsis diagnosis;  basal CRP <5.3mg∕dL;  score SAPS II <26 points) | **Model 2**  (medical admission;  sepsis diagnosis;  basal CRP 5.3-18.8mg∕dL;  score SAPS II 26-53 points) | **Model 3**  (medical admission;  sepsis diagnosis;  basal CRP >18.8mg∕dL;  score SAPS II >53 points) |
| --- | --- | --- | --- |
| Phenotype A  Cluster analysis | 172 (83%) | 35 | 0 |
| Phenotype B  Cluster analysis | 9 | 116 (70%) | 41 (25%) |
| Phenotype C  Cluster analysis | 10 | 54 (21%) | 192 (75%) |

CRP - C-reactive protein; SAPS II - Simplified Acute Physiology Score II
