# Supplementary figures and images for: Identification of distinct clinical phenotypes in mechanically ventilated patients with acute brain dysfunction using cluster analysis
Source: Medicine (Baltimore). 2020 May 1;99(18):e20041. doi: 10.1097/MD.0000000000020041 (PMC7440320; doi:10.1097/MD.0000000000020041)

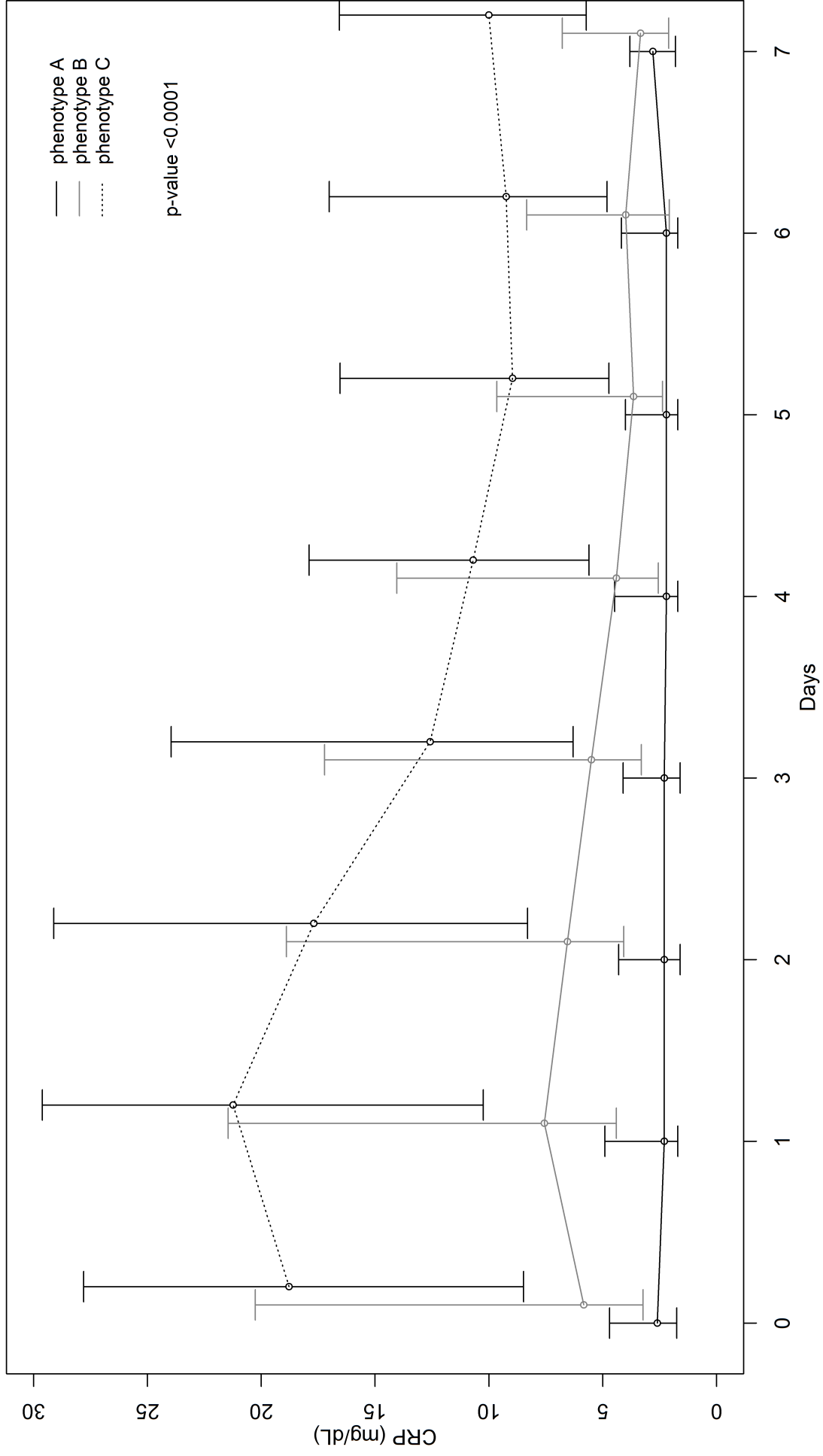

Supplement: Supplemental Digital Content [file medi-99-e20041-s005.pdf]
